# Bone marrow-derived mesenchymal stromal cells differ in their attachment to fibronectin-derived peptides from term placenta-derived mesenchymal stromal cells

**DOI:** 10.1186/s13287-015-0243-6

**Published:** 2016-02-11

**Authors:** Jan K. Maerz, Lorenzo P. Roncoroni, David Goldeck, Tanja Abruzzese, Hubert Kalbacher, Bernd Rolauffs, Peter DeZwart, Kay Nieselt, Melanie L. Hart, Gerd Klein, Wilhelm K. Aicher

**Affiliations:** KFO273, Department of Urology, University of Tübingen Hospital, Paul Ehrlich Str. 15, 72076 Tübingen, Germany; Center for Medical Research, Department of Medicine II, University of Tübingen, Tübingen, Germany; Interfaculty Institute of Biochemistry, University of Tübingen, Tübingen, Germany; BG Trauma Center Tübingen, University of Tübingen, Tübingen, Germany; Integrative Transcriptomics, Center for Bioinformatics, University of Tübingen, Tübingen, Germany

**Keywords:** Mesenchymal stromal cells, Cell attachment, Integrins, Bone marrow stem cells, Placenta stem cells

## Abstract

**Introduction:**

Human mesenchymal stromal cells (MSCs) can be isolated from different sources including bone marrow and term placenta. These two populations display distinct patterns of proliferation and differentiation in vitro. Since proliferation and differentiation of cells are modulated by cell–matrix interactions, we investigated the attachment of MSCs to a set of peptide-coated surfaces and explored their interactions with peptides in suspension.

**Methods:**

Human MSCs were isolated from bone marrow and term placenta and expanded. Binding of MSCs to peptides was investigated by a cell-attachment spot assay, by blocking experiments and flow cytometry. The integrin expression pattern was explored by a transcript array and corroborated by quantitative reverse transcription polymerase chain reaction and flow cytometry.

**Results:**

Expanded placenta-derived MSCs (pMSCs) attached well to surfaces coated with fibronectin-derived peptides P7, P15, and P17, whereas bone marrow-derived MSCs (bmMSCs) attached to P7, but barely to P15 and P17. The binding of bmMSCs and pMSCs to the peptides was mediated by β1 integrins. In suspension, expanded bmMSCs barely bind to P7, P13, P15, and less to P14 and P17. Ex vivo, bmMSCs failed to bind P7, but displayed a weak interaction with P13, P14, and P15. In suspension, expanded pMSCs displayed binding to many peptides, including P4, P7, P13, P14, P15, and P17. The differences observed in binding of bmMSCs and pMSCs to the peptides were associated with significant differences in expression of integrin α2-, α4-, and α6-chains.

**Conclusions:**

Human bmMSCs and pMSCs show distinct patterns of attachment to defined peptides and maintain differences in expression of integrins in vitro. Interactions of ex vivo bmMSCs with a given peptide yield different staining patterns compared to expanded bmMSCs in suspension. Attachment of expanded MSCs to peptides on surfaces is different from interactions of expanded MSCs with peptides in suspension. Studies designed to investigate the interactions of human MSCs with peptide-augmented scaffolds or peptides in suspension must therefore regard these differences in cell–peptide interactions.

**Electronic supplementary material:**

The online version of this article (doi:10.1186/s13287-015-0243-6) contains supplementary material, which is available to authorized users.

## Background

Multipotent mesenchymal stromal cells (MSCs) have been detected in and isolated from various tissues and niches. Today the main source for isolation of MSCs is the bone marrow in the iliac crest or in the femoral shaft [1, 2]. Closely related mesenchymal cells have been described in the white pulp of teeth [3–5] and as pericytes along the vasculature of adipose tissue [6–9], the endometrial and fetal parts of placenta [10–13], in muscle tissue [14, 15], and in inner organs [16, 17]. Furthermore, related cells have been isolated from umbilical cord blood [18] and peripheral blood [19, 20], urine [21], amniotic fluid and Wharton’s jelly of the umbilical cord [22, 23], and even from avascular tissue [24, 25]. The niches for these MSCs or MSC-like cells differ significantly in their mechanical and chemical composition. Bone marrow, for instance, is rather stiff (*E* ≈ 100 kPa), cartilage is considerably softer (*E* ≈ 30 kPa), muscle is quite elastic (*E* ≈ 12 kPa), and adipose tissue very flexible (*E* <10 kPa) [26]. Moreover, in bone marrow, type I, III, V and VI collagen, laminin isoforms containing the α4-, and α5-chains, fibronectin, and glycosaminoglycans dominate the stem cell niche [27–31], whereas pericytes of placenta are found in contact with laminin α2- and α5-chains and type IV collagen of the basal lamina and adjacent to fibronectin [32].

The MSCs from bone marrow (bmMSCs) express a significantly different transcriptome compared to MSCs from pancreas or placenta [16, 33]. Human bmMSCs differ in their growth kinetics and expression of integrin α4 from placenta-derived MSCs (pMSCs) [34]. Moreover, MSCs from adipose tissue express CD34 [35, 36], an antigen not found on bmMSCs [37–39]. Our recent studies are in line with these reports as we find significant differences between bmMSCs and pMSCs in their osteogenic differentiation capacities [40], expression of Runx2, WISP2, osteoglycin and osteomodulin [33], and expression of the stem cell markers alkaline phosphatase and CD146 [13].

Previously we investigated the binding and attachment of bmMSCs to proteins and peptides in comparison to fibroblasts [41]. There, fibroblasts differed from bmMSCs in both binding, as determined by the multiple substrate array technique [42], and short-term attachment [30]. Based on the fact that bmMSCs and pMSCs differed in their proliferation and differentiation capacities [13, 33, 34], and proliferation and differentiation of MSCs are modulated by the extracellular matrix and integrin signaling [43–50], we investigated the interaction of bmMSCs versus pMSCs with a set of peptides and the expression of integrins in more detail.

Our results suggest that i) bmMSCs and pMSCs differ significantly in their expression of integrins, and therefore in attachment to distinct peptides. In addition, ii) interactions of MSCs with peptides on a solid phase via attachment follow different kinetics or thermodynamics compared to interactions of MSCs with the same peptides in suspension, and iii) the expression of matrix-binding receptors on bmMSCs ex vivo seems be modulated by the in vitro culture condition. This may have interesting consequences when, for instance, attachment assays are performed in vitro to investigate the mobilization and migration of MSCs in the circulation and homing to specific niches.

## Methods

### Preparation of MSCs from femoral bone marrow and term placenta tissue

Aspirates from human femoral bone marrow (n = 15 patients, nine females, six males, mean age 67 years, average volume 12–15 mL) were obtained from the Clinic for Trauma and Restorative Surgery, BG Trauma Center Tübingen, University of Tübingen, after written and informed consent. The fraction of mononuclear cells was enriched by density gradient centrifugation and the cells were expanded as described recently [51]. Human term placenta was obtained from the Department of Gynecology and Obstetrics, University of Tübingen Hospital, from mothers undergoing planned Caesarean delivery after written and informed consent (n > 15 donors, mean age 34 years). The MSCs were isolated, purified and cultured in a good manufacturing practice (GMP)-compliant expansion medium as described recently [51]. Both types of MSCs were characterized according to the criteria defined by the International Society for Cellular Therapy by flow cytometry to confirm the expression of CD73, CD90, CD105, and CD146 as well as documenting lack or very low expression of CD11b or CD14, CD34, and CD45 (not shown) [33, 37, 40, 52]. The differentiation capacities of the MSCs investigated were confirmed in vitro by induction of osteogenic, adipogenic, and chondrogenic (bmMSCs), or adipogenic and chondrogenic differentiation (pMSCs), respectively [1] (not shown). Dermal fibroblasts were isolated from surgical waste from the skin of patients and expanded as described (n = 4, [41]). In some experiments MSCs were washed (2 × phosphate-buffered saline (PBS)) detached by mild proteolysis (5 min, 37 °C, Accutase^®^, PAA Laboratories), washed again, counted, and aliquots of 5 × 10^5^ viable MSCs were resuspended in 1 mL cold freezing medium (Dulbecco's modified Eagle's medium, 20 % fetal bovine serum (FBS), 10 % dimethyl sulfoxide), cooled further and stored in the gas phase of liquid N_2_ tanks. For additional studies, frozen aliquots of MSCs were rapidly thawed, washed twice with 25 mL medium, seeded in culture vessels, and cultured over night in GMP-compliant expansion medium. Then the cells were washed twice with PBS, detached by proteolysis (5 min, 37 °C, Accutase^®^), counted to confirm yield and viability, and utilized for the corresponding experiment. The study was approved by the Ethics Committee of the Medical Faculty of University of Tübingen (# 453/2011B02).

### Attachment of MSCs to proteins and peptides

Attachment of MSCs or fibroblasts to proteins and peptides immobilized on plastic surfaces was explored as described previously [30, 53, 54]. As substratum for cell attachment, bovine serum albumin (BSA) was activated by maleimide-ester, coupled with peptides, and separated from residual peptides by dialysis and gel filtration [41]. The peptide-modified BSA served as substrate for the cell attachment assays. Then cells were harvested as described above, viability was confirmed (>90 %, trypan blue dye exclusion) and 3 × 10^6^ cells were resuspended in 1.3 mL medium supplemented with 0.1 % PSA buffer (final concentration 0.9 % BSA/PSA) and ion-mix (final concentration 1 mM CaCl_2_,1 mM MgCl_2_, 0.025 mM MnCl_2_); 200 μL of this cell suspension was added to the substratum spots. After incubation at ambient temperature for 15 min, attachment of the cells was analyzed by microscopy. Cells not adhering to the spots were removed by washing (4 × PBS), and the cells attached recorded by dark-field and phase-contrast optics (Leica DM IRB, Leica Wetzlar Germany). Attachment of cells to laminin-111 (LM), fibronectin (FN) or BSA (all from Sigma-Aldrich) served as controls, respectively.

In a second line of experiments, attachment of MSCs to peptides or proteins was blocked by pre-incubation of 5 × 10^5^ MSCs per spot in 100 μL media with a function blocking monoclonal antibody (mAb) to human β1 integrin (anti-CD29, clone: 4B4, dilution 1:20, 4 °C for 30 min; Beckman Coulter Inc, USA). MSCs incubated with mAb to CD90 (clone: Thy1-A1, dilution 1:5, 4 °C for 30 min; R&D Systems) and mock-treated MSCs served as controls. Then the cells were added to the substratum spots, incubated, washed and recorded as described above.

To label cells by fluorescent dyes, 1 × 10^6^ cells in the second passage of in vitro culture were detached by mild proteolysis (5 min, 37 °C, Accutase^®^), washed twice with PBS and incubated with PKH26 (red label) or PKH67 (green label) as described [55] using standard reagent kits (Sigma-Aldrich, Taufkirchen, Germany). To confirm high viability of MSCs in the attachment assays, cells were loaded with Calcein-AM and Ethidium-homodimer (Live/Dead cell Staining Kit II, PromoCell, Heidelberg, Germany). Then attachment of cells was explored with the labeled MSCs as described above. Live cells emitting green fluorescence at 515 nm and dead cells emitting red fluorescence at 620 nm were recorded by a fluorescence microscope.

### Flow cytometry of MSCs after expansion in vitro and ex vivo

MSCs were expanded to the second passage, harvested as described above, collected in medium and washed with PBS to determine the cellular yield and viability in a hematocytometer by trypan dye exclusion. Cells (5 × 10^5^) were resuspended in fluorescence-activated cell sorting (FACS) buffer (500 mL PBS, 10 mL FBS, 0.5 % (w/v) NaN_3_, 372 mg ethylenediaminetetraacetic acid) and incubated on ice for 30 min with mAbs to different antigens at appropriate dilutions (Table 1). Then, the cells were washed twice, resuspended in FACS buffer again and explored by flow cytometry as described recently [52]. To test bmMSCs and pMSCs for peptide interactions, serial dilutions of fluorescein isothiocyanate (FITC)-labeled peptides (Table 2) were resuspended in FACS buffer and staining of MSCs was explored by flow cytometry as described above. Unstained MSCs and CompBeads (BD Biosciences, Heidelberg, Germany) served as controls [52]. To this end the CompBeads were resuspended in 200 μL FACS buffer for automatic compensation with the BD FACS Diva acquisition program (BD Biosciences). Data were processed and analyzed using FACS Diva and FlowJo 7.2.2 (Treestar Inc, Ashland, OR, USA) following recent guidelines [56]. Flow cytometry data were computed as geometric means of fluorescence intensity (MFI).

**Table 1:** List of reagents employed: monclonal antibodies for exploring mesenchymal stromal cells by flow cytometry

CD	Isotype	Clone	Label	Source	Company	Dilution
CD14	IgG2A	MφP9	FITC	Mouse	R&D Systems	1:5
CD34	IgG1	4H11	PE	Mouse	BioLegend	1:10
CD45	IgG1	MEM-28	PE	Mouse	BioLegend	1:5
CD73	IgG1	AD2	PE	Mouse	BD Bioscience	1:2.5
CD90	IgG2A	Thy1-A1	PE	Mouse	R&D Systems	1:5
CD105	IgG1	SN6	AF 488	Mouse	ABD Serotec	1:5
CD 271	IgG1	ME20.4-1.H4	APC/AF 647	Mouse	Miltenyi Biotec	1:5
CD29 / ITGB1	IgG1	MAR4	BV510	Mouse	BD Bioscience	1:10
CD49b / ITGA2	IgG2A	12 F1	PE-CF594	Mouse	BD Bioscience	1:10
CD49d / ITGA4	IgG1	9 F10	BB515	Mouse	BD Bioscience	1.10
CD49f / ITGA6	IgG2A	GoH3	PE-CF594	Rat	BD Bioscience	1:10
ITGA7	Polyclonal serum	ø	FITC	Rabbit	Biorbyt	1:10

**Table 2:** List of reagents employed: peptides

Peptide code	Amino acid sequence	Estimated molecular weight	Origin
2	GEFYF DLRLK GDK	1585 Da	Human collagen IV α1 chain
4	LAIKN DNLVY VY	1421 Da	Human laminin α4 chain G domain
7	WQPPR ARITG Y	1344 Da	Human fibronectin
13	AASIK AVAVS ADR	1257 Da	Human laminin α1
14	DVISL YNFKH IY	1509 Da	Human laminin α4 chain G domain
15	KREDV Y	808 Da	Synthetic peptide
16	EILDV (part of P17)	600 Da	Human fibronectin type III repeat
17	DELPQ LVTLP HPNLH GPEIL DVPST	2726 Da	Human fibronectin type III repeat

In other experiments cells were prepared ex vivo from fresh samples of bone marrow by Ficoll^®^ gradient centrifugation [51], washed, counted and directly stained with fluorescently labeled mAb to the MSC-specific antigen CD271 as described recently [38] (Table 1). Then, the cells were washed twice with FACS buffer and resuspended in FACS buffer with the diluted labeled peptides (Table 2), and incubated on ice. The bmMSCs were washed twice again and subjected to flow cytometry. Double labeled cells were analyzed in the dot blot mode with four quadrants. Unstained cells and cells incubated with anti-CD271 only served as controls and to set the gates.

### Differences in transcripts encoding integrins

To explore the differences in the expression of integrins between bmMSC and pMSC two data sets generated by gene array using the Affymetrix GeneChip technology were employed in this study as described recently [33]. Transcripts encoding integrins that were expressed significantly different in bmMSC versus pMSC were investigated further by quantitative reverse transcription polymerase chain reaction (qRT-PCR).

### qPCR of transcripts after reverse transcription

To enumerate the steady state mRNA expression in bmMSCs versus pMSCs, cells were harvested by Accutase® (PAA) and washed by cold PBS; 1 × 10^6^ MSCs were collected in 1.5 mL microtubes and mRNA was extracted (RNeasy, Qiagen, Hilden Germany). Reverse transcription was performed from 1 μg of total RNA (oligo-(dT)_n_priming, Advantage RT for PCR Kit, Clontech, Mountain View, USA) to generate the cDNA substrate for PCR. Gene-specific cDNA was enumerated by qRT-PCR (LightCycler, Roche, Mannheim, Germany) [57] using transcript-specific primers (MWG Eurofins, Ebersberg, Germany). Quantification of transcripts encoding GAPDH and PPIA served as references in each of the amplifications to normalize the amounts of the target gene by the FitPoint (ΔΔC_t_–) method [57]. The mean values of replicate experiments and standard deviations were calculated by Excel^®^ spread sheet software, and statistical significances between groups of data were computed with a two-sided paired Student’s *t*-test. Probability values (*p*) equal to or less than 0.05 were considered to be statistically significant.

## Results

### Attachment of human MSCs to proteins and peptides

**Fig. 1:**
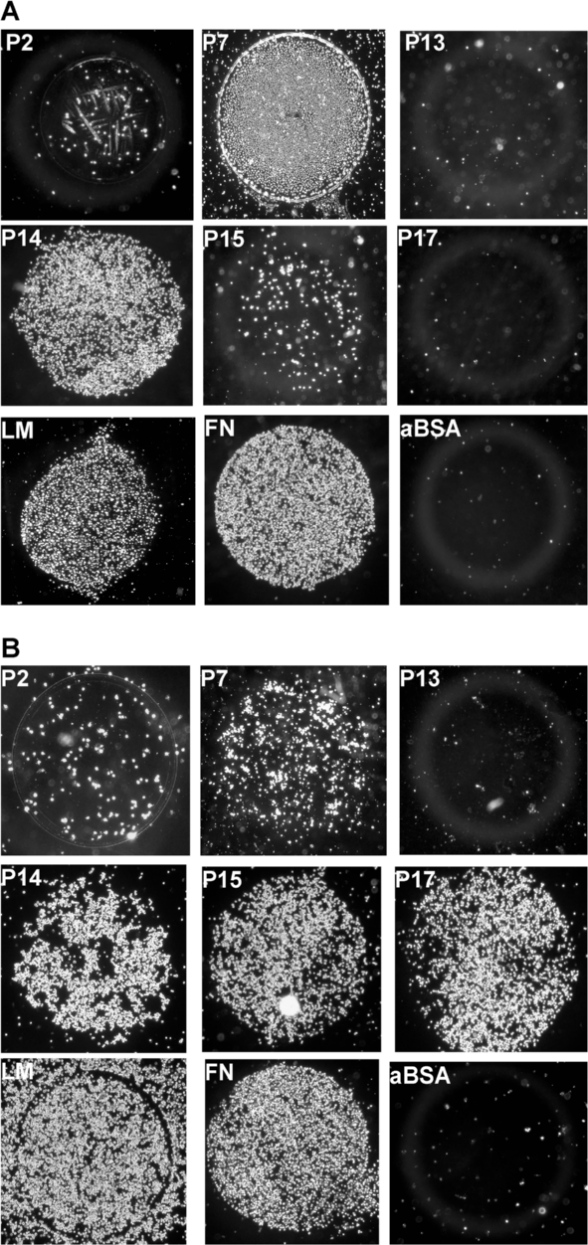
Attachment assay with human MSCs. Human MSCs from bone marrow (**a**) and term placenta (**b**) were incubated on spots coated with peptides P2–P17, laminin-111 (*LM*), or fibronectin (*FN*) as indicated. After 15 min of incubation non-adherent cells were washed away and MSCs attached are visualized by microphotography. Spots coated with activated bovine serum albumin (*aBSA*) served as controls

**Fig. 2:**
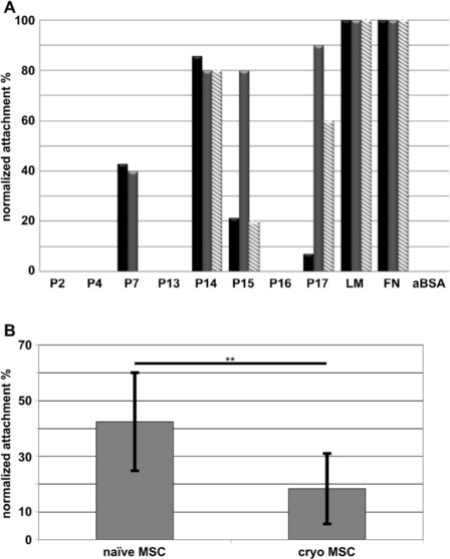
Overview on attachment patterns of human mesenchymal stromal cells (*MSCs*). **a** Human bmMSCs (n = 14 donors, *black bars*) or pMSCs (n = 10 donors, *grey bars*) were expanded to investigate their attachment to peptides P2–P17 as indicated. Attachment of MSCs to laminin-111 (*LM*) and fibronectin (*FN*) served as positive control (100 %), attachment to activated bovine serum albumin (*aBSA*) served as negative control (0 %). Attachment of human DF to the same substratum was tested in comparison to the MSCs (n = 5 donors, *hatched bars*). **b** Attachment of primary culture human MSCs to peptides P7, P14, P15, and P17 was tested prior to deep freezing (*naïve MSCs*) and compared to the same cells after cryopreservation and revitalization (*cryo MSCs*). Overall, naïve MSCs attached to the peptides at 43 ± 18 % mean efficacy compared to FN (=100 %). Cryopreservation caused a reduced overall attachment to these peptides of all MSCs investigated (19.2 ± 14.2 %). Thus, cryopreservation yielded a significant drop of attachment of human MSCs (n = 10, mean reduction 0.42 ± 0.20, *p* ≤ 0.0048)

Human bmMSCs and pMSCs were isolated, expanded and characterized to meet the International Society for Cellular Therapy criteria [37]. Second passage MSCs were harvested and added to peptide-coated dishes to compare their attachment to peptides in comparison to proteins (Fig. 1). Patterns of attachment of human bmMSCs and pMSCs differed considerably. While both types of MSCs attached well to LM-111 (all = 100 %), FN (all = 100 %), and the LM α4-chain-derived peptide P14 (≥80 %), most pMSCs attached well to FN-derived peptides P15 (80 %) and P17 (90 %), but only a few bmMSCs (20 % and 7 %, respectively) attached to peptides P15 or P17 (Fig. 2). Neither bmMSCs, pMSCs, nor dermal fibroblasts (DF) attached to peptides P2, P4, P13, P16, or to albumin (Fig. 2). The pMSCs and DF attached rather well to peptide P17 (Figs. 1 and 2). Peptide P16 is a part of P17 (Tables 1 and 2), but neither MSCs nor DF attached to P16 suggesting that this interaction between the cells and peptides is highly selective. Peptides P7, P15 and P17 are three different peptides derived from FN. The pMSCs preferably bound to P15 (80 %) and P17 (90 %) when compared to bmMSCs (20 % and 7 %; Fig. 2, Table 3). In contrast, about 40 % of both pMSCs and bmMSCs bound to FN-derived peptide P7. This suggested that MSCs isolated from different tissues express distinct patterns of receptors which interact with defined epitopes or peptides derived from extracellular matrix proteins.

**Table 3:** Comparison of cells attaching to peptides and proteins

	P2	P4	P7	P13	P14	P15	P16	P17	LM	FN	aBSA
bmMSC	0	0	42.8	0	84.5	20	0	7.1	100	100	0
pMSC	0	0	40	0	80	80	0	90	100	100	0
DF	0	0	0	0	80	19	0	90	100	100	0

Cells (bone marrow-derived mesenchymal stromal cells (bmMSC) from 14 donors, term placenta-derived mesenchymal stromal cells (pMSC) from 10 donors, dermal fibroblasts (DF) from 5 donors) were added to peptide-coated spots as listed, incubated for 15 min. and floating cells were washed away. The numbers in this table present the percentage of cell types attaching to a given peptide or protein. All cells attached well to laminin 111 (LM) and fibronectin (FN). They therefore served as positive controls (100 %). Attachment to activated bovine serum albumin (aBSA) was not observed and therefore served as negative control (0 %)

In addition, we investigated the attachment of MSCs to peptides P7, P14, P15, and P17 that facilitated sufficient binding of cells prior to deep freezing (naïve) and after cryopreservation (cryo) and revitalization (Fig. 2b). In all cases, attachment of cryopreserved bmMSCs and pMSCs was lower compared to the same cells prior to deep freezing, but it was not different in bmMSC compared to pMSC (*p* = 0.64). The mean normalized attachment of naïve MSC dropped 2.2-fold from 43 ± 18 % to 19.2 ± 14.2 % (n = 10, *p* ≤ 0.0048; Fig. 2b), suggesting that cryopreservation strongly influences the interaction between cells and the extracellular matrix.

**Fig. 3:**
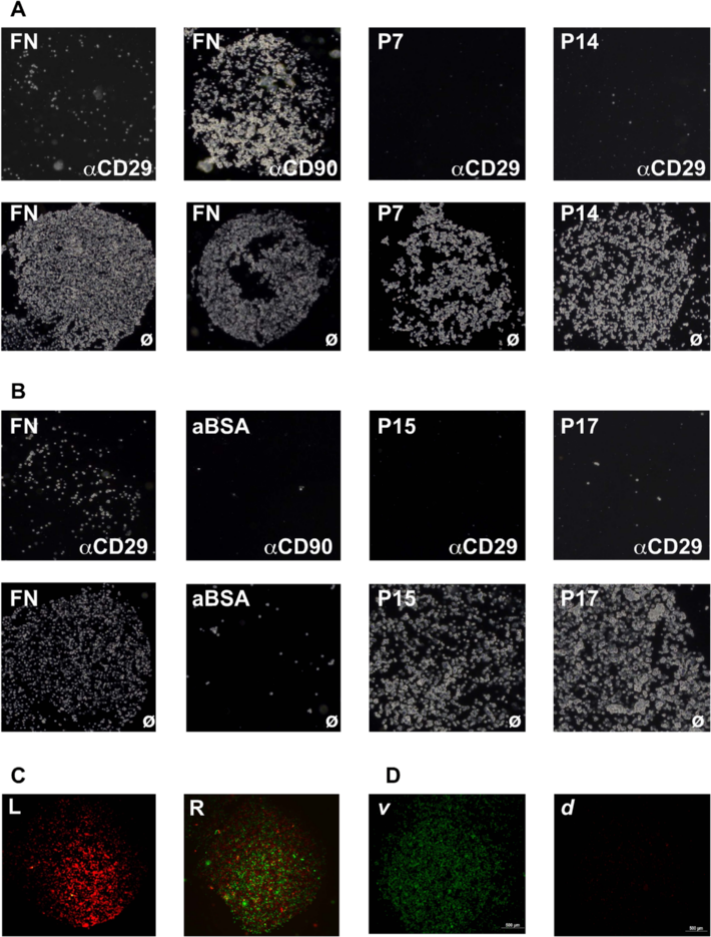
Contribution of integrins to protein- and peptide- dependent cell attachment. Human bmMSCs (**a**) or pMSCs (**b**) were pre-incubated with a function-blocking antibody to CD29 (integrin β1 chain) or remained untreated as indicated (ø). The cells were then incubated on peptide- or fibronectin (*FN*)-coated spots. Pre-incubation of bmMSCs (**a**) or pMSCs (**b**) with anti-CD29 mAb completely blocked the attachment of the cells, whereas untreated MSCs attached well to peptides or proteins. Pre-incubation of bmMSCs with anti-CD90 mAb failed to block attachment of the cells confirming the specificity of the blocking reaction (**a**, *upper right*). Incubation of pMSCs on activated bovine serum albumin (*aBSA*) did not cause unspecific binding of cells to this reagent (**b**). Human bmMSCs were labeled with PKH26 and attachment of PKH26-loaded cells to FN was confirmed (**c**, *left*). Human fibroblasts were labeled with PKH67, mixed 1:1 with PKH26-labeled MSCs and incubated on fibronectin (**c**, *right*). DF competed for binding sites and displaced the MSCs (**c**, *right*). Human bmMSCs were loaded with Calcein-AM and EthD-1 to discriminate viable cells (green cytoplasm, *v*) from dead cells (red nuclei, *d*) and added to LM-111-coated spots (**d**). The MSCs attached presented a bright green fluorescence indicating a high viability of the population studied (**d**, *v*). Only a few dead cells were observed (**d**, *d*)

To investigate the nature of the interactions between the human MSCs and the proteins or peptides, respectively, the cells were saturated with a function blocking mAb against the integrin β1 chain and then attachment to the MSCs binding proteins and peptides was explored (Fig. 3). Pre-incubation of the cells with a mAb to integrin β1 chain (i.e., anti-CD29) blocked the interaction between the bmMSCs and the substratum completely whereas pre-incubation of bmMSCs with an unrelated mAb (i.e., anti-CD90) did not interfere with their attachment (Fig. 3a). In analogy, pre-incubation of pMSCs with a mAb to the integrin β1 chain blocked attachment of the cells to FN and peptides P15 and P17 (Fig. 3b). To test if MSCs attached to maleimide ester-activated BSA in an unspecific manner, unmodified BSA and activated BSA were spotted onto glass slides and incubated with pMSCs. Binding of MSCs to activated BSA was not observed (Fig. 3b). This corroborated that the attachment patterns were generated by cell-to-peptide or cell-to-protein interactions and not by chemical binding of the cells to the remaining maleimide ester groups on activated BSA. To further confirm the specificity of the cell–substratum interaction, we incubated red labeled MSCs on FN or mixed green labeled DF with red labeled bmMSCs to compete for binding sites. Labeling bmMSC by PKH26 did not interfere with their attachment. The “green” fibroblasts reduced binding of the “red” MSCs in part (Fig. 3c). In other experiments the MSCs were labeled with Calcein-AM and Ethidium homodimer to stain viable and dead cells, respectively. The cells attached to LM-111 appeared green and only very few red nuclei were detected indicating a high viability of the MSCs employed in the attachment assays (Fig. 3d). We conclude that the distinct attachment patterns investigated in this study are mediated mainly by cell–peptide or cell–protein interactions involving β1 integrins.

### Interaction of human MSCs with peptides in suspension

**Fig. 4:**
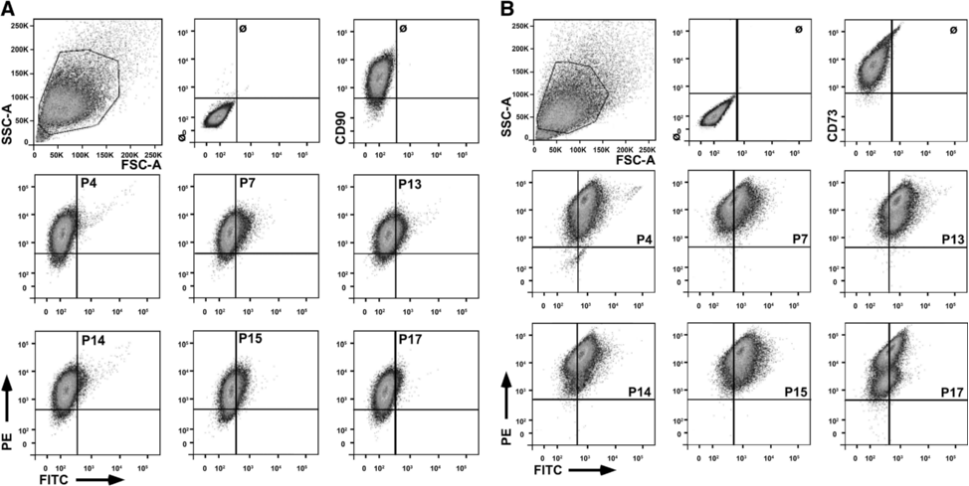
Interaction of peptides with human MSCs in suspension. Human bmMSCs (**a**) and pMSCs (**b**) were expanded to the second passage of in vitro culture, harvested by mild proteolysis, and stained with titrated amounts of FITC-labeled peptides. In addition, bmMSCs were counterstained with PE-labeled anti-CD90 (**a**), and pMSCs with PE-labeled anti-CD73 (**b**) to confirm their mesenchymal origin. Moderate binding of peptides P7, P13, and P15 was observed on bmMSCs (**a**). In contrast, pMSCs displayed a bright staining with all peptides investigated (**b**). *FITC* Fluorescein isothiocyanate, *FSC* Forward scatter, *PE* Phycoerythrin, *SSC* Side scatter

In general, MSCs are attached in vivo and in vitro to the extracellular matrix, to neighboring cells or another substratum. However, MSCs have also been detected in suspension in blood [18–20] and umbilical cord blood [58] and their numbers can be elevated in blood by hypoxia [59, 60]. They can be mobilized under stress conditions [61], but also engraft after intravenous injection functionally to the bone marrow [62]. This has been taken as evidence that MSCs may circulate in the blood and also home to their niche(s). We therefore also investigated the binding of peptides to expanded bmMSCs and pMSCs in suspension (Fig. 4). Binding of peptide P4 to bmMSCs was not observed in suspension. The peptides P7, P13, P14, P15, and P17 showed some binding to bmMSC in suspension, but the signal intensities recorded by flow cytometry (Fig. 4a) did not correlate with the attachment patterns observed (Fig. 2). In contrast to bmMSCs, all peptides stained pMSCs in suspension, and with P13, P14 and P17 pMSCs two distinct CD73^pos^ subsets were resolved (Fig. 4b). Depending on the protocol employed for isolation of MSCs from term placenta, the cell culture contains a blend of maternal and fetal MSCs and possibly smaller populations of other mesenchymal cells such a vascular smooth muscle cells. This may explain in part the differences in expression of CD73 in the pMSCs observed (Fig. 4b). Moreover, as seen with bmMSC, pMSC did not attach to the P13-coated surface (Fig. 1) but pMSC in suspension were stained with PE-labeled peptide P13 (Fig. 4b). This confirmed that bmMSCs and pMSCs display distinct interaction patterns with peptides. At the same time the results indicated that interactions of MSCs with peptides are variable when in suspension (Fig. 4) compared to interactions of the cells on a solid phase (Figs. 1, 2, 3).

### Interactions of peptides with bmMSCs ex vivo

**Fig. 5:**
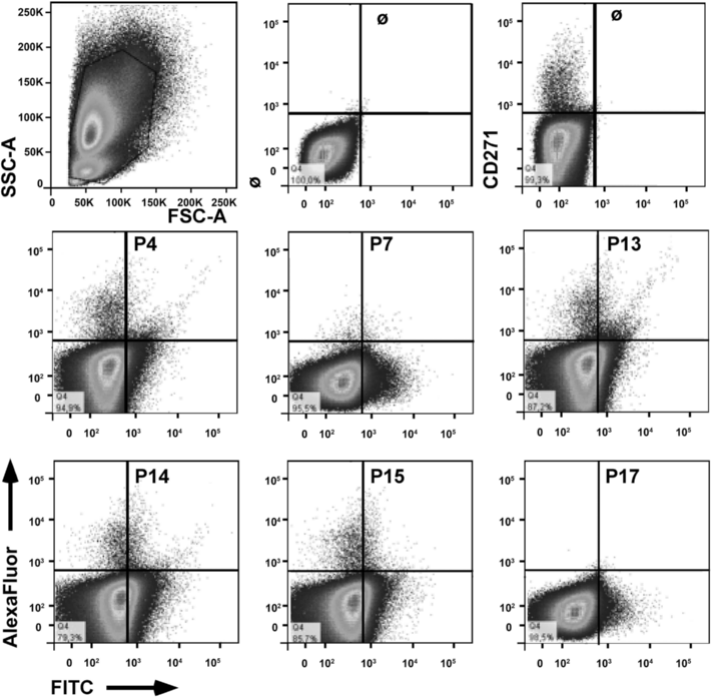
Interaction of peptides with human bmMSCs ex vivo. Mononuclear cells were purified from human bone marrow by Ficoll® gradient centrifugation, washed and incubated with peptides as indicated and counterstained with AF-647-labeled mAb to CD271, a marker for bmMSCs. Peptide P13 stained a small subset of CD271^pos^ bone marrow cells ex vivo, whereas peptides P4, P14, and P15 stained only very few CD271^pos^ cells. Peptides P7 failed to bind to CD271^pos^ cells, but all peptides interacted with CD271^neg^ bone marrow cells. *FITC* Fluorescein isothiocyanate, *FSC* Forward scatter, *SSC* Side scatter

Expression of cell surface proteins may be regulated by in vitro cell culture conditions. We therefore explored the binding of peptides to bmMSCs ex vivo (Fig. 5) in comparison to bmMSCs after expansion in vitro (Fig. 4). To discriminate bmMSCs from other cells in samples of human bone marrow ex vivo, the bmMSCs were labeled by a mAb to CD271 as described recently [38]. All peptides employed yielded at least some binding to mononuclear cells from human bone marrow by staining 1.5 % to 20 % of the CD271^neg^ population (Fig. 5, n = 3). Peptides P4, P13, P14, and P15 stained very few CD271^pos^ cells (about 0.2–0.5 % of all cells), whereas peptides P7 and P17 failed to bind to CD271^pos^ bone marrow-derived cells ex vivo (Fig. 5). This indicated that the expression of extracellular matrix receptors on MSCs is modified by the culture conditions utilized to expand the cells.

### Expression of integrins in bmMSCs and pMSCs in vitro

**Fig. 6:**
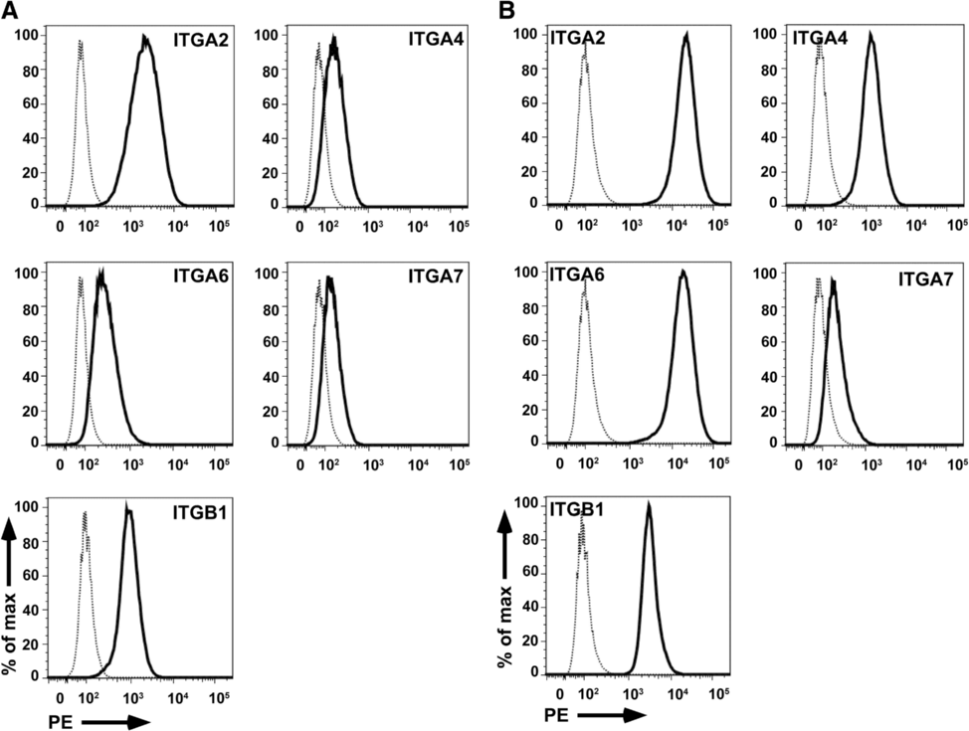
Detection of integrins on bmMSCs and pMSCs. Human bmMSCs (**a**) or pMSCs (**b**) were expanded to the second passage to investigate the expression of integrin α2, α4, α6, α7, and β1 by flow cytometry. Expression of integrin α2, α4, α6, and β1 was lower on bmMSCs compared to pMSCs (*solid histograms*). MSCs incubated with PE-labeled anti-Ig antibody served as control (*dotted histograms*). For integrin α7, no difference in expression levels was observed by flow cytometry. *PE* Phycoerythrin

Beta-1 integrins mediate the attachment of human MSCs to the proteins and peptides investigated in this study (Fig. 3). We therefore investigated the transcriptome of these populations to explore differences in the expression of integrins by gene array (Table 4). By this method the transcript amounts encoding collagen- and thrombospondin-binding integrin chain α2(β1), FN-binding integrin chain α4(β1) or α4(β7) and LM-binding integrin α6(β1) were significantly lower in bmMSCs compared to pMSCs, whereas transcripts of integrin chains α7(β1) and α11(β1) were higher in bmMSCs (*p* < 0.05). The differences in gene expression were confirmed by qRT-PCR with RNA extracted from MSCs of other donors. In bmMSCs integrin chains α2 (5.4-fold, statistically not significant) α4 (2-fold, *p* < 0.0006) and α6 (3.3-fold, *p* < 0.005) were expressed at a lower level, and integrin chains α7 (145-fold, *p* < 0.0001) and α11 (4.8-fold, *p* < 0.41, statistically not significant) were expressed at a higher level in bmMSCs compared to pMSCs (Table 4). We hypothesized that the differences in expression of integrin heterodimers contributed to the preferred in vitro attachment of pMSCs to peptides P15 and P17 when compared to bmMSCs (Fig. 2). We therefore explored the expression of integrin α-chains on bmMSCs and pMSCs by flow cytometry (Fig. 6). The expression of the integrin α2-, α4-, α6-, and β1-chains was lower on bmMSCs compared to pMSC whereas expression of integrin α7 revealed no differences in staining intensities (Fig. 6). This corroborated at the protein level the differences in expression of the integrin α2-, α4-, and α6-chains between human bmMSCs and pMSCs as observed on transcript levels by gene array and qRT-PCR (Gene Array data are available online, see Additional file 1).

**Table 4:** Expression of integrins in human MSCs in vitro

Transcript	Gene array (CFC)	R-NTR	
ITGA2	−2.5 (*p* < 0.05)	5.4-fold	(*p* = 0.063, s.n.s.)
ITGA4	−12.4 (*p* < 0.0001)	2-fold	(*p* < 0.0006)
ITGA6	−19.3 (*p* < 0.0001)	3.3-fold	(*p* < 0.005)
ITGA7	+6.8 (*p* < 0.0001)	0.007-fold	(*p* < 0.0001)
ITGA11	+4.6 (*p* < 0.0006)	0.2-fold	(*p* < 0.41, s.n.s.)

The table documents the differences in integrin transcript expression between bone marrow-derived mesenchymal stromal cells (bmMSCs) and term placenta-derived mesenchymal stromal cells (pMSCs) as evaluated by gene array (CFC; left, n ≥ 6 RNA samples each) or quantitative reverse transcription polymerase chain reaction (R-NTRs; right, n ≥ 4 cDNA samples each). CFC-values v > 1 denominate a higher, values v < 1 a lower expression in bmMSCs, respectively (CFC: combined fold change of transcripts enumerated by gene array, after evaluation with FDR corrections). *R-NTR* ratio of normalized transcript rates, *s.n.s.* statistically not significant

## Discussion

Our study provided evidence that MSCs can bind to small peptides in a specific way through integrins, that patterns of MSC–peptide interactions depend on the type of MSCs investigated, and that such interactions are modified by cell culture conditions. In addition, the attachment of cells to peptide-augmented surfaces differs from the patterns of staining of MSCs in suspension. Integrins are important receptors for anchoring cells in a tissue. They play an important role in pattern formation, tissue organization, differentiation, mobilization and homing of cells and many other physiological and pathological processes [48, 49, 63–66]. The composition of the extracellular matrix in a given tissue and even more in a (stem) cell niche is important to maintain the integrity of the tissue and at the same time its function [27, 67]. By choice of peptides such a niche or environment can possibly be mimicked on or in scaffolds to facilitate the homing or seeding of distinct cells in an in vivo situation.

We also provide evidence that fibroblasts, bmMSCs and pMSCs will attach to biomaterials modified with specific peptides in an ordered way. Therefore two- or even three-dimensional patterns of different cells and even closely related cells can be generated in vitro by modification of the scaffolds' surfaces with peptides. Blocking β1-integrins by a mAb abolished the attachment of the MSCs to both proteins and peptides. This confirmed that the peptide–cell interactions investigated in this study are highly selective and depend on β1-integrins.

However, the interaction of peptides with integrins yielded a higher dissociation constant K_D_ compared to the K_D_ measured for a naïve protein [68]. The RGD peptide–a popular motif used in many attachment assays–bound with a more than 50-fold lower affinity to its receptor (K_D_ = 1.7) compared, for instance, to fibrin, the prototype protein containing the RGD motif [68]. Moreover, attachment of cells to peptides by integrins depends not only on the avidity of the individual ligand (i.e., peptide/protein)–receptor (i.e., integrin) interaction, but also on the blend of different integrins and expression level of integrins on a given cell surface. Therefore, in the context of regenerative medicine or tissue engineering, a selectivity of peptide-modified scaffolds means a lower affinity of such a scaffold to the cell when compared to protein-coated surfaces. This can possibly be compensated for by distributing or patterning the peptides on scaffold surfaces according to the density of integrins on the cells [69]. Then cooperative effects will “glue” the cells to the scaffold.

As shown in one of our recent studies, expression of integrins on human MSCs is regulated by transforming growth factor beta (TGF-β) [53]. Others reported on the role of platelet-derived growth factor (PDGF), basic fibroblast growth factor (bFGF) and epidermal growth factor in regulation of integrins on MSCs or other cells [70–72]. These cytokines are found in the sera used to complement the growth medium for human MSCs [51], and their titers in media may differ from the concentrations recorded in situ. Therefore, expression of integrins on cells may differ in vivo compared to in vitro. We used GMP-compliant media enriched with human plasma and platelet extract for expansion of MSCs to keep our preclinical experiments close to a future clinical application [51]. Human platelet extract is a rich source for PDGF, TGF-β, bFGF and other factors [73]. This explains in part the differences observed in peptide binding between MSCs ex vivo and MSCs after expansion. This finding also suggests that binding assays performed with MSCs expanded in media containing xenobiotic serum cannot completely reflect the interaction of MSCs in situ. This problem applies to MSC-specific antigens used for ex vivo staining as well since expression of CD271 on human bmMSCs was elevated on cells ex vivo, but disappeared in primary cultured cells [13, 38]. Comparably, expression of CD34 on adipose tissue-derived human MSCs varies and depends on the cell culture conditions [7]. In addition, the concentration of growth factors detected in serum depends on the age of the donor [74]. Therefore, not only MSC proliferation, but also integrin expression, will depend on the quality of platelet extract used to enrich the MSC media. Expression of integrins on MSCs may change during in vitro culture and expression of integrin α6 chain was lost on human umbilical cord-derived MSCs after 5 passages expansion [75]. We used bmMSCs and pMSCs in their second passage of culture. Therefore loss of integrins on bmMSCs due to extended expansion in this time frame is not a likely event. A recent study on expression of integrins on human bmMSCs reported minimal or no detection of integrin α6, but intermediate to high expression of other integrins [76]. In contrast, we find some expression of integrin α6 chain on bmMSCs in our experiments. This seemingly conflict of results may be caused by the differences in preparation and maintenance of the bmMSCs since we used—unless specified differently—non-cryopreserved MSCs expanded in GMP-compliant medium for two passages. Danmark and colleagues used a commercial source for bmMSCs and therefore cryopreserved cells, and a commercial MSC medium of undisclosed composition [76]. Our data suggest that cryopreservation significantly reduced attachment of MSCs to peptides. These important differences in the experimental design may account for the different outcomes.

Moreover, short-term attachment of MSCs to surfaces is modulated in vitro by proteins found in the cell culture medium used. This is relevant when MSCs are in contact with surfaces that contain physiological ligands for high-affinity attachment of cells, such as RGD peptides. Biodegradable polymers such as lactate esters are widely used compounds for tissue engineering that do not contain natural cell binding motifs. Attachment of cells to such polymers depends therefore on the adsorption versus desorption of peptides or proteins to the polymer to which the cells then subsequently bind in the second place [77]. However, long-term binding of MSCs to any substratum in vitro and in vivo is not only modified by the composition of the pericellular milieu, but in addition modulated by proteins and extracellular matrix components produced by the MSCs themselves. For instance, integrin α5β3- and αvβ3-mediated binding of MSCs to FN triggers migration of the cells through PDGF-BB and PDGF-R signaling [70]. Binding of MSCs to FN or fibrin modulated their osteogenic differentiation in two- and three-dimensional cultures [78]. Laminin-322 modulated osteogenesis of MSCs [45], whereas type II collagen hydrogels together with TGFβ1 promoted chondrogenesis [79]. Our preliminary data suggest that pMSCs express, at least on a transcript level, less LAMA5, COL4A5 and COL13A1, but more COL5A3, COL14A1 and COL11A1 than bmMSCs. These differences in expression of extracellular matrix proteins by bmMSCs versus pMSCs add to the integrin-mediated differences discussed above by occupying the binding sites of their respective integrins. Moreover, MSCs also produce cytokines and growth factors including TGFβ1 thus modifying expression and conformation of integrins in an autocrine way [36, 53]. Therefore dissimilarities reported for attachment, migration, proliferation or differentiation of MSCs in different studies can be explained by the inconsistencies in the various protocols employed [45, 46, 48].

Covering β1 integrins on MSCs by a mAb blocked their migration to an infarcted heart [80], and others provided evidence for integrin α4 receptors in the context of MSC homing [81]. Expression of integrins differs between stromal cells from bone marrow and adipose tissue [36]. Our data complement these studies as we find differences in cell–peptide interactions observed between bmMSCs and pMSCs in vitro and between bmMSCs ex vivo and in vitro, and add to the evidence for the role of integrins in tissue and/or niche-specific homing of these cells.

## Conclusion

In summary, human MSCs derived from bone marrow or placenta maintain distinct differences in expression of integrins after expansion in fully GMP-compliant medium for at least two passages of in vitro culture. bmMSCs display differences in peptide binding ex vivo compared to bmMSCs in vitro. We conclude that studies investigating the interaction of MSCs with peptides are biased by the growth conditions utilized, and even GMP-compliant media enriched with human plasma and platelet extract do not reflect the milieu a MSC is exposed to in bone marrow or in other tissues in vivo. Studies involving peptide-augmented scaffolds designed for in vivo applications must take these differences in MSC–peptide interactions into account.

## Additional file

Additional file 1:
**Supplementary Table.** Expression values (log2) of 593 significantly differentially expressed genes between bone marrow-derived mesenchymal stromal cells (Bm) and term placenta-derived mesenchymal stromal cells (P). Normalized expression values for all 25 samples are shown. Rightmost two columns show the combined log2 fold change between the two groups (logFC) and the absolute fold change (CFC). (XLSX 248 kb)

## Abbreviations

bFGF: Basic fibroblast growth factor
bmMSC: Bone marrow-derived mesenchymal stromal cell
BSA: Bovine serum albumin
DF: Dermal fibroblasts
*E*: Elastic modulus
FACS: Fluorescence-activated cell sorting
FBS: Fetal bovine serum
FITC: Fluorescein isothiocyanate
FN: Fibronectin
GMP: Good manufacturing practice
LM: Laminin
MIF: Mean intensity of fluorescence
mAb: Monoclonal antibody
MSC: Mesenchymal stromal cell
PBS: Phosphate-buffered saline
PDGF: Platelet-derived growth factor
pMSC: (term) Placenta-derived mesenchymal stromal cell
PSA: Phosphate buffer solubilized albumin
qRT-PCR: Quantitative reverse transcription polymerase chain reaction
RGD: arginine–glycine–aspartic acid (peptide motif)
TGF-β: Transforming growth factor beta
